# Management of Deep Brain Stimulator Battery Failure: Battery Estimators, Charge Density, and Importance of Clinical Symptoms

**DOI:** 10.1371/journal.pone.0058665

**Published:** 2013-03-11

**Authors:** Kaihan Fakhar, Erin Hastings, Christopher R. Butson, Kelly D. Foote, Pam Zeilman, Michael S. Okun

**Affiliations:** 1 Departments of Neurology and Neurosurgery, University of Florida Center for Movement Disorders and Neurorestoration, Gainesville, Florida, United States of America; 2 Departments of Neurology & Neurosurgery, Medical College of Wisconsin, Milwaukee, Wisconsin, United States of America; University Hospital La Paz, Spain

## Abstract

**Objective:**

We aimed in this investigation to study deep brain stimulation (DBS) battery drain with special attention directed toward patient symptoms prior to and following battery replacement.

**Background:**

Previously our group developed web-based calculators and smart phone applications to estimate DBS battery life (http://mdc.mbi.ufl.edu/surgery/dbs-battery-estimator).

**Methods:**

A cohort of 320 patients undergoing DBS battery replacement from 2002–2012 were included in an IRB approved study. Statistical analysis was performed using SPSS 20.0 (IBM, Armonk, NY).

**Results:**

The mean charge density for treatment of Parkinson’s disease was 7.2 µC/cm^2^/phase (SD = 3.82), for dystonia was 17.5 µC/cm^2^/phase (SD = 8.53), for essential tremor was 8.3 µC/cm^2^/phase (SD = 4.85), and for OCD was 18.0 µC/cm^2^/phase (SD = 4.35). There was a significant relationship between charge density and battery life (r = −.59, p<.001), as well as total power and battery life (r = −.64, p<.001). The UF estimator (r = .67, p<.001) and the Medtronic helpline (r = .74, p<.001) predictions of battery life were significantly positively associated with actual battery life. Battery status indicators on Soletra and Kinetra were poor predictors of battery life. In 38 cases, the symptoms improved following a battery change, suggesting that the neurostimulator was likely responsible for symptom worsening. For these cases, both the UF estimator and the Medtronic helpline were significantly correlated with battery life (r = .65 and r = .70, respectively, both p<.001).

**Conclusions:**

Battery estimations, charge density, total power and clinical symptoms were important factors. The observation of clinical worsening that was rescued following neurostimulator replacement reinforces the notion that changes in clinical symptoms can be associated with battery drain.

## Introduction

Deep brain stimulation (DBS) is a commonly performed surgical intervention that utilizes an implantable pulse generator (IPG), known as a neurostimulator, as a waveform generator and power source. The neurostimulator controls the flow of current to specific brain regions through an attachment to an implantable DBS lead. Each DBS lead has multiple contacts, and therefore many possible parameter configurations [1]. The optimization of possible settings, which may number into the thousands when considering the range of pulse widths, frequencies, amplitudes and configuration of anodes and cathodes, can provide a critical determinant for therapeutic success or failure [2]. The potential optimization parameters include changes to the configuration of the active electrodes, adjustments in voltage, current changes, lengthening or shortening of the pulse width, and changes in the frequency of impulses.

There are many factors that may influence battery drain. These factors include neurostimulator manufacturing tolerances, battery usage, battery chemistry, tissue impedance [3], interpolation error, usage patterns, and self-discharge [4]. These factors cannot unfortunately be taken into account by any single and available battery estimation technique. There is a battery status indicator that DBS programmers can check in clinic to monitor the remaining battery life of the IPG, but it is unknown whether this indicator is accurate. The battery status indicator was designed to be an estimation of the remaining battery voltage, and the range varies based on each specific battery type. An off the shelf Medtronic Soletra battery begins its life with a voltage typically between 3.69 and 3.72 volts, and it reaches its end of life (EOL) at voltages in the general range of 2.5 volts. The Medtronic Kinetra has a starting battery voltage of 3.2 and typically reaches EOL in the range of 1.97 volts. Since ideal DBS treatment involves replacement of the IPG battery prior to depletion, battery estimators have become an important part of management; however the existing voltage estimators have generally been poor clinical predictors for bedside management. In addition to avoiding total battery failure by preemptively replacing batteries, we examined whether there was a change in clinical symptoms occurring prior to battery replacement that is resolved following battery replacement. If such a relationship could be established, it would emphasize the importance of early replacement and accurate battery estimation.

The techniques to estimate neurostimulator battery life provide only approximations, and the available tools have been referred to as “estimators,” rather than calculators [4]. The management of battery life for neurostimulators should also take into account potentially critical fluctuations in clinical symptoms that may be attributable to battery drain. Previously, we published web-based and smartphone applications capable of rendering general DBS battery estimates. We also provided with these estimators an important accompanying algorithm for DBS battery management [4]. In this paper we apply the available DBS battery estimator techniques (UF and Medtronic helpline) to a large cohort of DBS patients, and also analyze the factors potentially important to battery drain, and to DBS battery management (e.g. disease type, battery type, current drain). In the current investigation, we focused on the following four research questions:

1. What were the relationships between charge density, total power, battery life, and battery type (Soletra, Kinetra) in the overall sample?
2. Did charge density and total power vary by diagnosis, and did the relationships between charge density and battery life, and total power and battery life, differ across diagnoses?
3. Was there a relationship between actual battery life and the UF estimator, the Medtronic helpline-predicted battery life, or battery status indicator in the entire sample, within each diagnosis group, within each charge density group (high, medium and low), within each total power group (high, medium and low), and across battery types (Soletra, Kinetra)?
4. Does the relationship between the UF estimator, the Medtronic helpline estimator, or the battery status indicator and actual battery life differ based on reason for battery change (e.g. battery failing, symptom worsening) across the entire sample?

We expect that the battery status indicator as reported by the DBS programmer would be a poor predictor of battery life. IPGs contain high-quality lithium-based batteries that have pretty flat discharge curves, which behave more like an ideal power supply, but make it difficult to predict remaining life. For monopolar stimulation with a single cathode we expect that increases in voltage or pulse width will reduce battery life because more charge is injected per pulse. Likewise, we expect that increasing frequency will reduce battery life because more pulses are delivered per second. We expect that using multiple cathodes will reduce battery life because a roughly equal amount of power is being delivered from each cathode (this is only true for voltage-controlled systems such as the Soletra and Kinetra). Lastly, we also expect that higher impedances will reduce battery life because more power is being dissipated in the tissue.

## Methods

### Participants

The dataset consisted of 320 DBS battery replacements drawn from a period between 2002–2012. Participants had diagnoses of Parkinson’s disease (PD) (n = 131), Dystonia (n = 110), Essential Tremor (ET) (n = 55), or Obsessive Compulsive Disorder (OCD) (n = 15). Nine subjects were excluded from analyses because of overlapping diagnoses. The general patient characteristics have been summarized in Table 1. Significance of all group differences were examined using separate univariate ANOVAs with diagnosis as the independent variable and age, battery life, and disease duration at battery replacement as independent variables.

**Table 1:** General Patient Characteristics.

	Age at Replacement (Years)	Battery Life (Days, Years)	Disease Duration at Battery Replacement(Days, Years)
**Parkinson’s Disease**	63.7, SD = 8.58	1321 (3.63 years), SD = 440.8	5730.2 (15.7 years), SD = 1673.4
**Dystonia**	37.3, SD = 16.03	782.9 (2.2 years), SD = 439.8	7032.4 (19.3 years), SD = 3706.7
**Essential Tremor**	62.9, SD = 19.68	1290.9 (3.54 years), SD = 606.5	9701.3 (26.7 years), SD = 6184.2
**OCD**	34.8, SD = 1.58	650 (1.79 years) SD = 264.5	7864.9 (21.6 years), SD = 752.7
**Total**	53.1, SD = 18.99	1090 (2.99 years), SD = 538.4	6995.4 (19.2 years), SD = 3830.8

### Procedure

The study protocol was approved by the Institutional Review Board (IRB) at the University of Florida (UF). The study utilized data from the UF INFORM database of DBS patients. There was also a separate IRB-approved chart review. We requested a waiver of informed consent from the UF IRB because data was obtained from several hundred patients who underwent DBS surgery with battery replacement. This request was approved by the UF IRB. The patients were de-identified in our analysis and many had moved away from the area or were no longer alive. Patients had already given approval on informed consent documentation to have their charts reviewed for purposes of research. Data collected included the diagnosis, date of diagnosis, IPG model, last known DBS parameters prior to battery replacement, charge density, total charge, UF estimate of battery life, Medtronic company helpline (Minneapolis, MN) estimate of battery life, battery status indicator, any documented symptom worsening prior to battery replacement, any documented symptom change following battery replacement, battery parameters at the time of replacement, whether or not the patient slept with the device on, dates of battery implantation and replacement, and the exact patient age at the time of battery replacement.

The average charge density was calculated using the following equation:
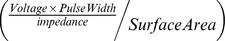



All patients had Medtronic 3387 or 3389 DBS leads implanted. The electrode contacts on these leads are 1.5 mm tall and 1.27 mm diameter, providing a surface area of 0.06 cm^2^ that was used in all charge density calculations. This equation assumes that the stimulation pulse is a square wave, which is a reasonable approximation at low pulse widths [5]. Additionally, it assumes that the voltage output of the DBS programmer matched the voltage output of the IPG, which is a reasonable approximation for frequencies of 130 Hz or lower [6].

Following a general analysis of charge density, a secondary analysis was performed by splitting the charge density groups into three roughly equivalent tertiles (low, medium, high). This analysis was based on two equal cut-points that were determined by SPSS 20.0 using the range of charge densities present in our dataset. Splitting the data into roughly equivalent thirds, the lowest third (n = 102) had charge densities of 0–6.82 µC/cm^2^/phase (actual range 1.37–6.82 µC/cm^2^/phase), the middle third (n = 102) had densities of 6.83–11.38 µC/cm^2^/phase (actual range 6.83–11.38 µC/cm^2^/phase), and the upper third (n = 102) had densities of 11.39+ µC/cm^2^/phase (actual range 11.39–45.8 µC/cm^2^/phase). An identical procedure was performed using total charge, instead of charge density. Total charge is calculated by multiplying stimulation voltage by pulse width and then dividing by impedance. The only difference between charge density and total charge is that the latter does not take into account surface area of the lead, and we would expect the two values to be highly correlated. Splitting the data into roughly equivalent thirds, the lowest third (n = 102) had charges of 0–0.42 µC (actual range 0.03–0.42), the middle third (n = 102) had charges of 0.42–0.76 µC (actual range 0.42–0.76), and the upper third (n = 102) had charges of 0.76 µC and up (actual range 0.76–2.75). All relevant analyses were run separately (due to high multicollinearity between the two variables, *r* = .96, p<.001) and analyses used charge density and total charge. There were no notable differences in the results between charge density and total charge, and only charge density numbers have been detailed in this paper.

In addition to examining charge density and total charge, the following analyses also include an investigation of total power, which we expected to be most strongly correlated with battery life since it takes into account frequency, unlike charge density. Total power was calculated using the following formula:
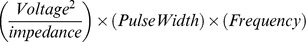



For Soletras this calculation was straightforward, but for Kinetras the pulse width was not always identical on both leads. Therefore, in Kinetras the power was calculated for each lead separately and then they were added together.

Total power was strongly positively correlated with charge density (r = .85, p<.001) and total charge (r = .88, p<.001), but also incorporated frequency so is important to consider. Splitting the data into roughly equivalent thirds, the lowest third (n = 102) had total power of 0–171.45 µW (actual range 12.33–171.12), the middle third (n = 102) had total power of 171.46–384.62 µW (actual range 172.12–383.07), and the upper third (n = 102) had total power of 384.63 µW and up (actual range 385.41–1752.49).

## Results

### Descriptive Statistics

Overall, subjects with PD were older than those with Dystonia and OCD, and had a longer battery life (all p<.001). Subjects with Essential Tremor also were older than those with Dystonia and OCD and also had longer battery life (all p<.001). Subjects with Dystonia and OCD did not differ based on age or battery life (both p = 1.00), and subjects with PD and Essential Tremor also did not differ (both p = 1.00). Of all the diagnoses, subjects with Essential Tremor had the longest disease duration at the time of their battery replacement, and this difference was significant when compared with PD and Dystonia (both p<.001) but not OCD (p = .478). Disease duration at battery replacement was significantly longer for Dystonia patients than for PD patients (p<.05) (see Table 1 for the summary of the means and standard deviations).

### Research Question 1

We first explored relationships between charge density, total power, battery life, battery type, and diagnosis. A Pearson correlation was calculated between charge density and battery life suggesting that in the entire sample there was a significant relationship between charge density and battery life (r = −.59, p<.001), such that the higher the charge density, the shorter the battery life. Total charge and charge density were highly correlated, r = .96, p<.001. Total charge was also significantly related to battery life at r = −.59, p<.001. Total power was significantly related to battery life as well, r = −.64, p<.001, such that the lower the power, the longer the battery life.

There were two IPG models used during the study period: Soletra (N = 288) and Kinetra (N = 32). In the PD group 125 received Soletra batteries and 6 received Kinetra. In the Dystonia group 90 received Soletra batteries and 20 received Kinetra. In the Essential Tremor group, 49 received Soletra batteries, and 6 received Kinetra. In the OCD group 15 Soletra batteries were implanted and 0 Kinetra. A Chi-Square analysis revealed that the group differences were significant, χ^2^(3) = 13.79, p<.003, such that the Dystonia group was more likely to receive the Kinetra battery than the PD group, χ^2^(1) = 10.97, p<.05, or OCD, χ^2^(1) = 24.44, p<.001 group (http://bitnos.com/info/chi-square-post-hoc-test). The Essential Tremor group was not significantly different than any other group with regard to battery type. An independent sample t-test showed that the two battery types did not differ on either charge density, t(304) = −.48, p = .64, or battery life, t(45.20) = 1.56, p = .13. However, the two battery types did differ on total power such that Soletra batteries had lower power (M = 348.56, SD = 323.74) than Kinetra batteries (M = 581.73, SD = 433.06), t(35.16) = −2.95, p<.01. This difference in power can be attributed to the fact that the Kinetra supports two leads, rather than one in the Soletra.

### Research Question 2

To examine the relationships between charge density, total power and diagnosis, a multivariate Analysis of Variance (MANOVA) was run using diagnosis (PD, Dystonia, ET, and OCD) as the predictor, and charge density and total power as the outcome variable. Pillai’s trace was used, as the sample sizes across diagnoses groups was unequal and this estimation is considered most robust [7]. The resulting MANOVA was significant, F(6,586) = 27.79, p<.001, η_p_^2^ = .20. Tests of between-subjects effects revealed that both univariate effects of charge density, F(3, 293) = 33.63, p<.001, η_p_^2^ = .26, and total power were significant, F(3, 293) = 63.14, p<.001, η_p_^2^ = .39. The mean charge density for PD was 7.17 µC/cm^2^/phase (SD = 3.82), for Dystonia was 17.50 µC/cm^2^/phase (SD = 8.53), for Essential Tremor was 8.30 µC/cm^2^/phase (SD = 4.87), and for OCD was 18.01 µC/cm^2^/phase (SD = 4.35). Mean total power for PD was 219.72 µW (SD = 150.77), for Dystonia was 593.11 µW (SD = 422.53), for Essential Tremor was 293.41 µW (SD = 243.6), and for OCD was 513.54 µW (SD = 185.76). Bonferroni-corrected post hoc tests revealed that differences between PD and ET did not reach significance with regard to charge density, but Dystonia was significantly different from both PD and ET (both at p<.001), as was OCD (both at p<.01). OCD and Dystonia did not significantly differ from each other. An identical pattern was found in Bonferroni-corrected post hoc tests for total power.

To test whether the relationship between battery life and charge density was similar across diagnoses, linear regressions were run for each diagnosis (except OCD, due to the small sample size), using battery life as the outcome, and charge density as the predictor. Each regression controlled for age at the time of replacement, and whether the patient turned the battery off while sleeping. All three regressions were significant at p<.001, suggesting a significant portion of battery life was explained by the predictors for each disorder. Additionally all standardized betas associated with charge density were significant at p<.001, such that the higher the charge density, the shorter the battery life for all three disorders. For PD, the standardized beta associated with charge density was −.27 (p<.001). For Dystonia, the standardized beta associated with charge density was −.51 (p<.001). For Essential Tremor, the standardized beta associated with charge density was −.55 (p<.001). The age at time of replacement, and whether the patient turned the battery off to sleep, only emerged as important predictors of battery life for the Dystonia group. Specifically, those who turned their battery off to sleep had significantly better battery life (b = .18, p<.05), and those who were younger at the time of battery replacement tended to have better battery life (b = −.16, p = .065). These relationships did not emerge for PD or Essential Tremor.

Due to the high correlation between total power and charge density (r = .85, p<.001), the multicollinearity assumption required for multiple regression was violated and so total power could not be included in the previous models. To test whether the relationship between total power and battery life was similar across diagnoses, separate linear regressions were run for each diagnosis (except OCD, due to the small sample size), using battery life as the outcome, and power total as the predictor. As in the previous analyses, each regression controlled for age at the time of replacement, and whether the patient turned the battery off while sleeping. All three regressions were significant at p<.001, suggesting a significant portion of battery life was explained by the predictors for each disorder. As was the case for charge density, all standardized betas associated with total power were significant at p<.001, such that the higher the total power, the shorter the battery life for all three disorders. For PD, the standardized beta associated with charge density was −.52 (p<.001). For Dystonia, the standardized beta associated with total power was −.62 (p<.001). For Essential Tremor, the standardized beta associated with charge density was −.57 (p<.001). Neither the age at time of replacement, nor whether the patient turned the battery off to sleep, emerged as significant predictors in these regressions.

A univariate ANOVA was run to examine battery life using diagnosis, charge density group and total power group (low, medium, and high, see methods section for description), and all interactions as the predictors and the battery life as the outcome. The resulting AVOVA was significant, F(21,273) = 15.12, p<.001, η_p_^2^ = .54. Tests of between subjects effects showed that diagnosis, F(3,273) = 3.17, p<.05, η_p_^2^ = .03, charge density group, F(2, 273) = 3.06, p<.05, η_p_^2^ = .02, and total power, F(2, 273) = 11.56, p<.001, η_p_^2^ = .08, were all significantly associated with battery life. Bonferroni-corrected post hoc tests showed that PD and ET did not significantly differ with regard to battery life, and neither did Dystonia and OCD. However, PD and ET both differed significantly on battery life from Dystonia and OCD such that the former two disorders had longer battery lives than the latter two disorders (all p<.05). The mean battery life for PD was 1235.92 days (SE = 54.52), for ET was 1231.51 (SE = 85.45), for Dystonia was 1004.07 (SE = 54.66), and for OCD was 431.5 (SE = 166.42). Charge density group was also associated with battery life. Bonferroni-corrected post hoc tests revealed that all three charge density groups differed from one another at p<.001 such that the shortest battery life was found in the highest charge density group (M = 790.56 days, SE = 66.74), the longest battery life was found in the lowest charge density group (M = 1571.68 days, SE = 86.64), and the medium charge density group fell in the middle with regard to battery life (M = 1082.75, SE = 55.47). Finally, total power group was associated with battery life. Bonferroni-corrected post hoc tests showed that all three groups differed significantly from each other (p<.05 for all) such that the shortest battery life was found in the highest power group (M = 671.52 days, SE = 56.30), the longest battery life was found in the lowest power group (M = 1423.41 days, SE = 65.02), and the middle power group fell in the middle with regard to battery life (M = 1157.88 days, SE = 74.95). Thus, in response to the first two research questions identified in the Methods section, there was a significant negative relationship between charge density (as well as total power) and battery life in our sample, and this relationship held across all diagnoses.

### Research Question 3

**Figure 1:**
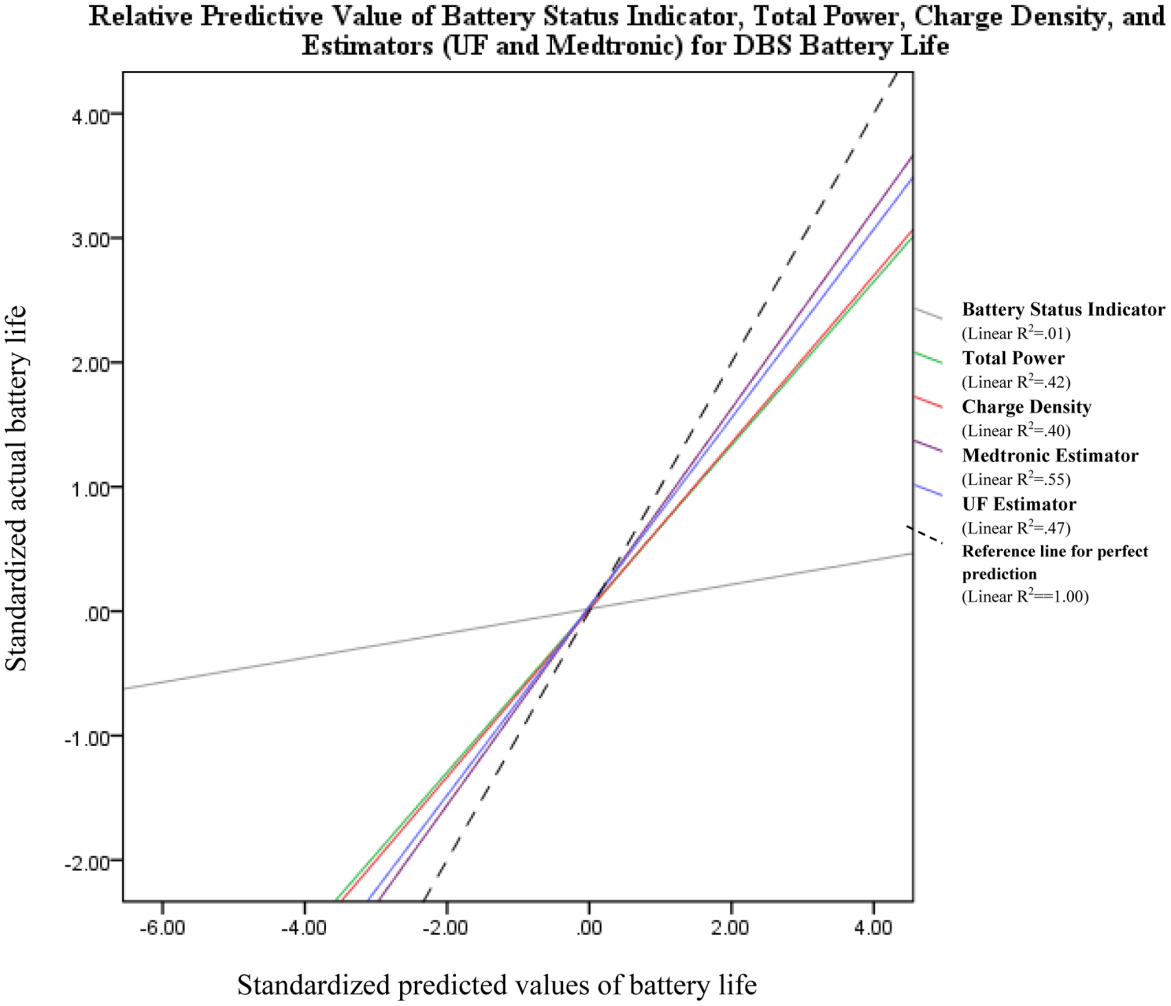
A comparison of the predictive value for DBS battery life. Both the X and Y axis represent standardized values because independent variables were on different scales, so the numbers plotted above are Z-scores. The UF estimator and the Medtronic helpline were best at predicting actual battery life, followed by total power and charge density, and finally by the battery status indicator. The dotted line represents a reference line for the perfect prediction of battery life by any technique employed.

The remaining two research questions focused on the UF estimator and Medtronic helpline predictors, and how they related to other variables in our dataset. First, a Pearson correlation was run between both the predicted battery life and the actual battery life. The results revealed that both the UF estimator (r = .67, p<.001) and Medtronic helpline (r = .74, p<.001) predictions of battery life were positively associated with the actual battery life. This result suggested that both predictors were accurately capturing battery life. Battery status indicator was also significantly associated with battery life, though the magnitude of this relationship was smaller than that of either predictor (r = .14, p<.05). Separate regressions were run for five different predictors (UF estimator, Medtronic helpline estimation, charge density, total power, and battery status indicator) to obtain a comparison of how each variable was related to the actual battery life (the outcome variable in each regression). Because the battery status indicator and charge density were represented on different numeric scales than the estimators, the standardized predicted value of battery life for each variable was used to plot against the actual battery life. The results are summarized in Figure 1. The figure illustrates that the estimators were best at predicting actual battery life (Medtonic linear R^2^ = .55, UF Estimator linear R^2^ = .46), followed by the charge density (R^2^ = .40), and finally by the battery status indicator (R^2^ = .02).

Next, the relationships between the predicted and the actual battery life and charge density and total power groups (low, medium, and high, see methods section for description) were investigated to ascertain whether the relationships differed based on charge density or total power. Separate Pearson correlations were run for each charge density and total power group, between the battery life and the predictors. The results showed that the UF estimator was significantly correlated to battery life for all three charge density groups (low r = .47, p<.001, medium r = .31, p<.001, high r = .35, p<.001) and all three power level groups (low r = .43, p<.001, medium r = .35, p<.001, high r = .22, p<.001), such that the higher the predictor value, the higher the battery life. The Medtronic helpline predictor was also significantly positively related to battery life for all charge density groups (low r = .49, p<.001, medium r = .56, p<.001, high r = .68, p<.001) and all three power level groups (low r = .47, p<.001, medium r = .51, p<.001, high r = .58, p<.001). The battery status indicator was not related to battery life in the low (r = .10), middle (r = .09), or high charge density group (r = .03). Similar results were found when the sample was split by total charge rather than by charge density. When the predictors were compared to battery life with battery type taken into consideration, both the UF estimator and the Medtronic helpline predictor were significant for the Soletra (r = .70 and r = .75, respectively, both p<.001). Only the Medtronic helpline predictor was significant for the Kinetra (r = .54, p<.001), however the sample size was comparatively very small for the Kinetra. Next, the relationships between predicted and actual battery life and diagnosis (PD, Dystonia, and Essential Tremor; the sample size for OCD was too small to include) were investigated. Separate Pearson correlations were run for each diagnosis between battery life and the predictors. The results revealed that the UF estimator was significantly related to battery life for PD (r = .53, p<.001), Dystonia (r = .61, p<.001), and Essential Tremor (r = .81, p<.001). The Medtronic predictor was also significantly related to battery life for PD (r = .59, p<.001), Dystonia (r = .83, p<.001), and Essential Tremor (r = .81, p<.001). The battery status indicator was not related to battery life in the PD (r = .12), Dystonia (r = .09), or Essential Tremor groups (r = .09). The battery status indicator was related to battery life in the Soletra battery (r = .17, p<.001), but not the Kinetra (r = −.21). Interestingly, the magnitude of the relationship between the battery status indicator and the Kinetra battery was larger than that of the relationship between battery status indicator and Soletra battery, but in the opposite direction, and this was non-significant (likely due to the smaller sample of subjects who received the Kinetra battery).

### Research Question 4

**Figure 2:**
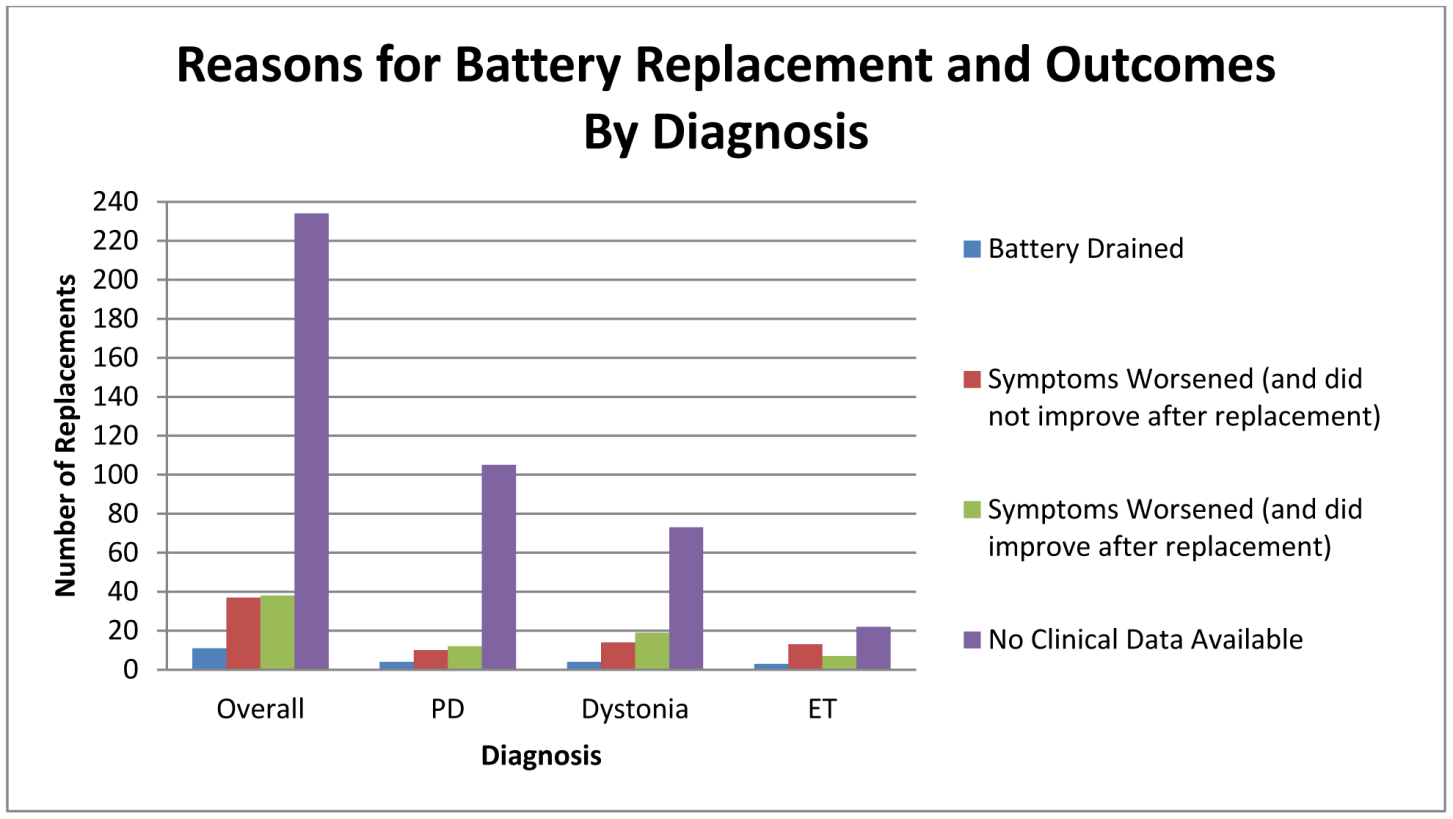
In each diagnosis group, total battery failure (battery drained) was the least likely reason for replacement. Essential Tremor was the only group in which symptom worsening without symptom improvement was more common than symptom worsening with improvement following a replacement surgery. The no clinical data represents a group of patients where the medical records described symptom worsening as the cause of battery replacement but did not clearly document the patient’s subjective improvement or worsening of symptoms following a battery replacement.

To investigate the question regarding the relationship between the reason for battery change (shown in Figure 2) and each of the battery estimators, Pearson correlations were performed. In 11 cases, the battery was changed because it was completely drained, and could not be accessed by the DBS programmer. For this group, neither the estimator nor the battery status indicator were significantly related to battery life (UF estimator r = .13; Medtronic estimator r = .19, battery status indicator r = .26). It is important to note, however, that this sample size was very small. For 7 of these cases, there was symptom improvement following battery replacement, suggesting that the drained battery affected clinical symptoms. The other 4 did not have data available for this variable.

In 75 cases, the battery was changed because disease symptoms were worsening. In 37 of these cases, the symptoms did not improve after the battery change, suggesting that disease worsening (rather than battery drain) was the underlying issue for the preponderance of symptoms. For these cases, both the UF estimator and the Medtronic helpline were significantly correlated with battery life at the time of replacement (r = .87 and r = .89, respectively, both p<.001). The battery status indicator was not related to battery life (r = −.003). In 38 of these cases, the symptoms improved after a battery change, suggesting that the battery was likely responsible for the symptom worsening. For these cases, both the UF estimator and the Medtronic helpline were significantly correlated with battery life (r = .65 and r = .70, respectively, both p<.001). The results suggested that, regardless of the reason for battery replacement, both estimators accurately captured battery life at time of replacement. The battery status indicator was not significantly related to battery life in this group (r = .19).

## Discussion

The results from this study revealed that the DBS neurostimulator battery life was reasonably predicted by either the UF estimator, or the estimation provided by the Medtronic company support line. The neurostimulator battery life was negatively correlated to charge density, suggesting that charge density is a potentially important factor useful for neurostimulator management. Both estimation techniques were more accurate than using a simple charge density, supporting their value in the preemptive replacement of DBS batteries. The improvement in symptoms in 38 patients following battery replacement underscores the importance of monitoring and the potential importance of pre-emptive battery changes.

One interesting, and unexpected finding was the relationship between battery life and charge density being strongest in the highest charge density group. This finding can be possibly explained by several clinical factors. First, lower charge densities likely impart a longer battery life. A longer battery life also will expose and individual patient to the potential for more optimization and therefore more changes in settings. Multiple setting changes over time can introduce error. Additionally, the longer a battery remains in service, the more factors can potentially alter the predictability of a single measurement. Factors important to the neurostimulator battery life include battery chemistry, local tissue impedance fluctuations, interpolation error, usage patterns, and self-discharge [4].

Aside from the estimation techniques, charge density was the variable that emerged as the single most important for predicting battery life, though it should be noted that charge densities in this study were observed to have a very wide range (1.37 to 45.8 µC/cm^2^/phase). Charge density represents the total charge per pulse divided by the surface area of a given DBS contact. Charge density can account for the parameters of stimulation, and also for the impedance, making it a unique and important measurement. Estimation of neurostimulator battery life in the dual-channel Kinetra battery subtype, however, introduced a new and important factor into the charge density calculation. Although the same equation listed in the methods was utilized, the two channels in the Kinetra are known to be interleaved in time, and to not be overlapping. The total charge of the each of the two channels therefore has to be calculated independently, and then added together. The total charges can then be summed and divided by the contact surface area to derive the correct total charge density. It was not surprising that the Kinetra estimations were less accurate than the Soletra, as Kinetra has been noted by clinicians to be more difficult to gauge in terms of battery life (author observations). Previous groups have documented differences between Soletra and Kinetra waveforms under identical stimulation settings [6].

Patients utilizing DBS are typically offered a vague estimate of their IPG battery life at each of their clinical visits. This estimate is often rendered simply by using the patient diagnosis. For Parkinson’s disease and Essential Tremor many clinicians commonly estimate 3–5 years [8], [9], and for dystonia 1–3 years [10]. Anticipating battery failure is a critical clinical issue, since sudden interruption of DBS therapy can result in medical emergencies in dystonia [11], PD [12], and OCD [13]. Some of the newer battery types (Medtronic SC and PC) have been built with an Elective Replacement Indicator (ERI) that also provides a general warning in the last few weeks of a neurostimulator’s battery life. These batteries however, have not been on the market long enough to test in the manner we tested Soletra and Kinetra for this current paper.

A variety of factors, in addition to stimulation parameters and impedance, are known to impact battery life. Many of these factors are difficult to take into consideration in a battery estimation (e.g. turning devices off during sleep, electromagnetic interference) [14], [15]. Though common battery estimators may not capture these factors, the UF estimator does have settings to account for sleep and cycling.

Since the ultimate goal of DBS therapy is to minimize symptoms and to improve quality of life, replacing neurostimulators prior to total failure would be the optimal course for treatment. Pre-emptive replacement will, however, skew battery estimations since estimations are designed to predict when the neurostimulators will reach total failure. This was a definite and unavoidable limitation of our study. Neurostimulator battery life is typically measured by the interval between implantation and replacement, regardless as to whether an individual experiences a total battery failure. In clinical practice, batteries are replaced for many reasons including: total battery failure, significant declines in a battery status indicator, or because of symptom worsening (with or without a decline in a battery estimate). General battery status voltage indicator estimations in our series were found to not be as accurate as the two estimators or charge density. A limitation in our series was that only a few batteries reached total failure (n = 11). Another limitation was that although the UF estimator possessed the capability to account for parameter changes over time, since the study was focused on comparing the two estimation methods, only the last documented DBS parameters were utilized.

It was interesting that in this cohort, we observed large numbers of patients with PD and ET who following battery replacement did not experience improvement in symptoms. These findings would suggest disease progression [16]. However, a reasonable sized group of patients did report symptom improvement, further reinforcing the importance of considering patient symptoms in the suggested algorithm for battery replacements [4].

In summary, both available DBS battery estimator techniques (UF and the Medtronic helpline) proved useful when applied to a large cohort of DBS patients. Charge density emerged as an important factor associated with battery life. Total battery failures were uncommon in this cohort, suggesting that the UF practice was generally effective in efforts for pre-emptive replacement prior to total failure. Drawing from the group of patients that experienced symptom worsening prior to replacement, approximately equal numbers of patients had symptom improvement following battery replacement. The improvements following battery replacement that were observed in many patients in this cohort suggest that neurostimulator issues could underpin clinical worsening. Battery estimates may be less predictable in the Kinetra battery neurostimulator subtype as compared to the Soletra. The observation of clinical worsening that could be rescued following neurostimulator replacement in 38 cases reinforces the notion that clinical symptoms can be associated with battery drain.
